# Experimental investigation of short-term warming on arsenic flux from contaminated sediments of two well-oxygenated subarctic lakes

**DOI:** 10.1371/journal.pone.0279412

**Published:** 2022-12-21

**Authors:** Brittany C. Astles, John Chételat, Michael J. Palmer, Jesse C. Vermaire

**Affiliations:** 1 Geography and Environmental Studies, Carleton University, Ottawa, Canada; 2 National Wildlife Research Centre, Environment and Climate Change Canada, Ottawa, Canada; 3 North Slave Research Centre, Aurora Research Institute, Yellowknife, Canada; Universidade de Lisboa Instituto Superior Tecnico, PORTUGAL

## Abstract

Legacy arsenic (As) contamination from past mining operations remains an environmental concern in lakes of the Yellowknife area (Northwest Territories, Canada) due to its post-depositional mobility in sediment and potential for continued remobilization to surface waters. Warmer temperatures associated with climate change in this subarctic region may impact As internal loading from lake sediments either by a direct effect on sediment porewater diffusion rate or indirect effects on microbial metabolism and sediment redox conditions. This study assessed the influence of warmer temperatures on As diffusion from contaminated sediment of two lakes with contrasting sediment characteristics using an experimental incubation approach. Sediments from Yellowknife Bay (on Great Slave Lake) contained predominately clay and silt with low organic matter (10%) and high As content (1675 μg/g) while sediments of Lower Martin Lake had high organic matter content (~70%) and approximately half the As (822 μg/g). Duplicate sediment batches from each lake were incubated in a temperature-controlled chamber, and overlying water was kept well-oxygenated while As flux from sediment was measured during four weekly temperature treatments (7°C to 21°C, at ~5°C intervals). During the experiment, As diffused from sediment to overlying water in all cores and temperature treatments, with As fluxes ranging from 48–956 μg/m^2^/day. Arsenic fluxes were greater from Yellowknife Bay sediments, which had higher solid-phase As concentrations, compared to those of Lower Martin Lake. Short-term warming did not stimulate As flux from duplicate cores of either sediment type, in contrast with reported temperature enhancement in other published studies. We conclude that warmer temperatures were insufficient to strongly enhance sediment As diffusion into overlying oxic waters. These observations are relevant for evaluating climate-warming effects on sediment As mobility in subarctic lakes with little or no thermal stratification and a well-oxygenated water column.

## Introduction

Arsenic (As) is a naturally occurring metalloid that is toxic and a known carcinogen to humans and other organisms [1]. Arsenic is mobilized within the environment through anthropogenic activities such as ore processing, disposal of mine waste, fossil-fuel combustion, and pesticide application [2]. Arsenic contamination is commonly released during mining and the roasting of gold ores that contain As-bearing sulphides [3,4]. The roasting and processing of As-rich ores generates mineralogical changes, which can alter As solubility, bioaccessibility and toxicity [5,6], creating a potentially hazardous contaminant once it enters the environment.

In Yellowknife, Northwest Territories, Canada, atmospheric emissions of As from roasting of As-bearing ores at local gold mines resulted in As contamination in the surrounding environment. Sediments of local lakes close to the historical mine roasters commonly have As concentrations > 500 μg/g dry weight [7–10], which is far above the Canadian environmental guideline of 17 μg/g for the protection of aquatic life [11]. Ore roasting took place at Giant Mine from 1949 to 1999, to transform gold-bearing arsenopyrite into gold-bearing iron (Fe) oxides, so that gold could be extracted via cyanide leaching [6]. The roasting process released As trioxide (As_2_O_3_) directly into the atmosphere via stack emissions. Con Mine, another local gold mine that operated in Yellowknife from 1938 to 2003, roasted ore intermittently until 1970 and was an additional source of As contamination within the area. It has been estimated that ~ 20,000 tons of As_2_O_3_ were released from ore roasting by Giant Mine between 1949 and 1958 [12]. In 1958, a baghouse facility was constructed at Giant Mine to collect the As_2_O_3_ dust from the roaster emissions and direct atmospheric emissions were greatly reduced [12]. In addition, tailings from Giant Mine were piled on the shore of Yellowknife Bay on Great Slave Lake from 1948–1951, resulting in transport of As contaminants directly into Yellowknife Bay. Furthermore, untreated effluent was released into Yellowknife Bay from Baker Creek until the construction of a wastewater treatment plant around 1980. Baker Creek continues to load approximately 1 tonne of As per year to Yellowknife Bay.

Several studies have assessed the environmental controls governing the distribution and mobility of As in lakes of the Yellowknife area [8–10,13–15]. There is seasonal variation of As mobility from lake sediments that is influenced by redox conditions and winter hydrology [13,14]. Research conducted by Andrade et al. [15] on the biogeochemical cycling of As contaminated sediments in Yellowknife Bay determined that mine-impacted lake sediments act as both a source and sink for As, depending on the seasonal redox conditions. Palmer et al. [13] showed that the mobility of As depended on physical characteristics of the lake and biogeochemical processes within the lake, with lake depth being an important factor. Shallower lakes showed greater seasonal change compared to deeper lakes because lakes less than 4 m depth typically developed anoxic conditions through the full water column under ice cover during winter, which promoted the upward migration of As from contaminated lake sediments [13]. Palmer et al. [14] also showed that sediments were a source of As to the overlying water during oxygen-depleted periods in the winter when there was no water inflow to the lake. When there was hydrological connectivity in winter, the As cycling changed due to the maintained presence of dissolved oxygen in the water column, which inhibited the upward movement of As from sediment [14].

Previous studies have shown that sediment temperature and organic matter (OM) content are key factors in controlling the mobilization of As from lake sediments to overlying water [16,17]. Microbial decomposition of organic carbon requires oxygen or other electron acceptors, which in turn alters redox conditions within sediments along the redox cascade to progressively reducing conditions. The reductive dissolution of Fe-oxide minerals, with adsorbed or co-precipitated As, can release As from the sediments into the overlying water [16,18,19]. Temperature has been shown to impact the mobility of As from sediments via increased microbial activity, which alters the position of redox boundaries within sediments [13,16,20]. Organic matter can also alter As mobility within the sediment environment through complexation with organic ligands [8,17,21].

With warming temperatures of lake water and sediment expected in the subarctic associated with climate change, it is critical to understand temperature effects on the mobility of legacy As contamination within lake sediments of the Yellowknife area. The objective of this study was to determine the impact of temperature on sediments from two lakes with different sediment and OM characteristics. The effect of warming on As diffusion from sediment was tested under controlled laboratory conditions for these two sediment types. We predicted that warmer temperatures would increase the rate of the As flux from contaminated sediments into the overlying water column, and that OM rich sediments from Lower Martin Lake would respond more to warming due to the greater potential for microbial metabolism.

## Materials and methods

### Environmental background and study site

**Fig 1:**
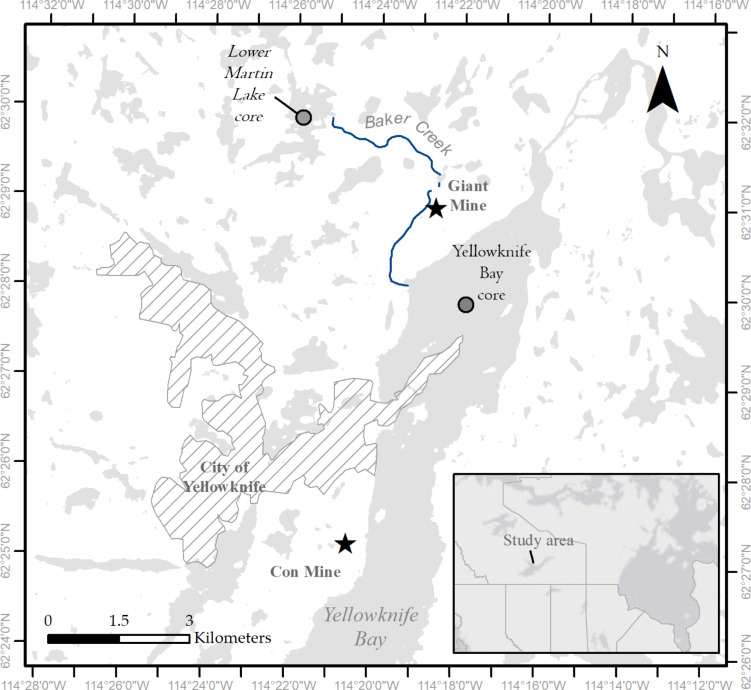
Map of the study area. Approximate locations where the sediment cores were collected from Lower Martin Lake and Yellowknife Bay in relation to the closed Giant Mine and Con Mine, and the capital of the Northwest Territories, Yellowknife, Canada. Spatial data presented in the figure are licensed under an Open Government Licence–Canada from the National Hydro Network (NHN) dataset https://open.canada.ca/data/en/dataset/a4b190fe-e090-4e6d-881e-b87956c07977.

Lake water and sediment were collected from two lakes contaminated by mining pollution near Yellowknife, Canada (**Fig 1**). Lower Martin Lake and Yellowknife Bay on Great Slave Lake are in the southern area of the Slave Geological Province of the Canadian Shield. The climate of the area is continental subarctic, having long winters with a mean January temperature of -26°C, and short warm summers with a July mean temperature of 17°C [22]. Lake ice (which typically reaches a thickness of 1–1.5 m over the winter) melts between mid-May to early June, depending on the timing of spring warming and lake size, and ice forms again on lakes in October or November, making for a short (~4 month) open water season.

Great Slave Lake is a large cold-water, oligotrophic lake [23], ranked the world’s ninth largest by surface area (28,568 km^2^). Yellowknife Bay on the north arm of Great Slave Lake (62° 30’ 36.7"°N, 114° 19’ 8.0°W), has a surface area of 20 km^2^ and a maximum depth of 15 m at the north end (where the sediment sampling occurred). Metal(loids) such as As and antimony, as well as metals, were deposited into the north end of Yellowknife Bay from mining activities at Giant Mine via Baker Creek [7]. Chételat et al. [7] determined the peak sediment concentrations of As in Yellowknife Bay were 741–4560 μg/g, which is approximately 180 times greater than pre-mining levels. At the north end of Yellowknife Bay where the sediment cores for this study were collected, As concentrations in surface waters were ~ 3 μg/L, which is estimated to be three to five fold greater than the measured background. Arsenic in the surface water of Yellowknife Bay was primarily inorganic As [7].

Lower Martin Lake is a small lake with organic-rich sediment, a maximum water depth of 2.9 m, and an area of 1.21 km^2^ (62° 30’ 43.2"°N, 114° 25’ 15.6"°W) [14]. Lower Martin Lake is within 5 km of the decommissioned roaster of Giant Mine and is a natural waterbody situated outside the mine boundaries. It is part of the Baker Creek watershed, which has a drainage area of about 155 km^2^ and flows through the Giant Mine property before finally discharging into Yellowknife Bay [14]. Lower Martin Lake is located downwind of the Giant Mine under predominant easterly winds [13], and arsenic contamination was from aerial deposition of ore roasting emissions that occurred at Giant Mine during operations [9]. Lower Martin Lake acts as a control on the fluxes of legacy mining contamination entering Great Slave Lake from the Baker Creek system [14]. Surface water As in Lower Martin Lake ranges between 20 and 150 μg/L (primarily as inorganic As), depending on dissolved oxygen conditions in overlying waters [9,14], and is an order of magnitude greater than Yellowknife Bay.

### Sediment and lake water collection

*In situ* sediments of Lower Martin Lake and Yellowknife Bay have previously been well characterized, including detailed profiles of porewater element concentrations, redox boundary depth, As mineralogy, and particle size. During the open-water season of both lakes, sharp peaks of porewater As concentrations are observed around 1–3 cm depth in the sediment, decreasing upward toward the sediment-water interface [7,14,15]. The sediment redox boundary is likely around that depth (~1–3 cm) based on geochemical indicators in the sediment profiles including dissolved oxygen, porewater concentrations of inorganic As species (arsenite, arsenate), dissolved Fe, and the importance of Fe (oxy)hydroxides as a mineral host in the surface layer [7,9,14,15,24]. Below that boundary, reductive dissolution of As_2_O_3_ and As-bound Fe (oxy)hydroxides act as sources of As to porewaters while the precipitation of As sulphides acts as a sink [9,14,15,24]. Lower Martin Lake sediments are composed largely of OM (69–76% by weight) [14], including benthic algal mat and detrital material, while Yellowknife Bay sediments are largely inorganic material, predominately silt (44 ± 15%) with lesser amounts of clay (25 ± 17%) and sand (31 ± 20%) [25].

Field collections of environmental samples were permitted under Aurora Research licence numbers 16366 and 16463. Duplicate sediment cores from Yellowknife Bay and Lower Martin Lake were collected using a gravity corer at a water depths of ~ 8 m and ~ 2 m, respectively. The top 15 cm of sediment from the Yellowknife Bay cores was extruded and transported unfrozen as a mixed bulk sample. The sediment cores from Lower Martin Lake were organic-rich, which allowed the cores to be frozen intact on the day of collection, without swelling and cracking of the core tubes. In contrast, the high clay and low OM content of the Yellowknife Bay sediment did not allow for freezing. Previous attempts showed freezing excessively altered the clay sediment layers. Surface water from the lakes was also collected for use in the incubation experiment following filtration with a high capacity 0.45 μm filter, acid-washed Teflon tubing and a peristaltic pump. Filtered lake water was stored at 4°C in acid-washed 1–4 L HDPE bottles.

Sediments and water were shipped to National Wildlife Research Centre in Ottawa to carry out the laboratory experiments. The frozen Lower Martin Lake cores were thawed and left intact in the original core tubes used for their collection. In contrast, mixed sediment from the top 15 cm of the Yellowknife Bay cores was added to the bottom 10 cm of two new core tubes. Sediment from both lakes was incubated in plastic core tubes 8.6 cm in diameter with filtered lake water added over top.

### Experimental design

The sediment mesocosms were incubated in a temperature-controlled growth chamber. The growth chamber lights were illuminated for 16 hours a day to mimic diurnal light exposure for the Lower Martin Lake during early summer, which receives natural light under *in situ* conditions due to the lake’s shallow depth. Black plastic was used to entirely cover the Yellowknife Bay sediment (to mimic dark conditions in offshore deep-water) and partially covered the Lower Martin Lake cores to prevent illumination of sub-surface sediment (**S1 Fig in S1 File**). This difference in plastic covering did not affect the temperature treatments for individual cores (based on temperature sensor measurements in each core). Oxygen bubblers were placed in each core to slowly aerate and mix the overlying water, which is representative of natural (well-mixed) conditions in the study lakes. The bubbler flow was sufficiently low and above the sediment surface to avoid disturbance to the sediments throughout the experiment. A porewater port was drilled into the sediment cores, at ~ 1 cm below the sediment-water interface and sealed so that porewater samples could be extracted using a syringe.

Initially, the sediment mesocosms were incubated in a growth chamber at 5°C for a 2-week period to allow the sediments and overlying water to equilibrate. After the 2-week equilibration period, the overlying water from the cores was gently removed using a large syringe and replaced with 1.5 L of fresh filtered lake water that was collected during fieldwork. The water was extracted and replaced slowly to avoid disturbing the top layer of the sediment as much as possible. The cores were then given a 48-hour period to allow any disturbed sediment to settle at 5°C.

The experiment was conducted over a 30-day period with 4 consecutive temperature treatments at approximately 5, 10, 15 and 20°C. The same sediments were gradually warmed over time to simulate a progression in temperature change, as would occur *in situ* in the lakes, rather than comparing stand alone sediment incubations at a single temperature. Mesocosm temperatures increased by ~ 2°C above programming during operation of lights in the growth chamber (**S1 Table in S1 File**). Each treatment was approximately one week in length with 3 to 5 sampling days. Surface waters were sampled for concentrations of As, Fe and manganese (Mn) to estimate metal(loid) efflux into the overlying water column of each core with each temperature treatment. At the beginning and the end of each temperature treatment, porewaters were collected for As, Fe and Mn (Lower Martin Lake sediments only), and surface water for dissolved nitrogen (DN), dissolved organic carbon (DOC), and sulphate (SO_4_^2-^). Dissolved nitrogen and DOC were measured for indicators of OM breakdown and microbial activity. Samples extracted for SO_4_^2-^ concentration were measured to assess oxidation of sediment sulphides. For sampling at the end of the third temperature treatment of 15°C, the temperature of the growth chamber was raised to 20°C a day before the porewater, DN, DOC and SO_4_^2-^ water samples were collected. Those results were still used and assumed to be representative of measurements for the end conditions of the 15°C temperature treatment.

### Surface water measurements

Sensors to measure temperature, pH, and oxidation-reduction potential (ORP) were calibrated and connected to a laptop for continuous readings at 30-minute intervals using the 8-Channel Monitor/Data Logger (MM-PIT-8S) (EA Instruments Ltd., Wembley, Middlesex, United Kingdom). The pH electrode (EAI pH2011) was calibrated at the start of the experiment using standard solutions of pH 4, 7 and 10. The ORP electrode (ORP-31 C sensor) was calibrated at the start of the experiment using Zobell standard solution. Port holes were drilled into the cap of each core tube for sensor installation from the top of the core into overlying water and for extracting water samples with Rhizon samplers.

Water samples (5 mL) were collected for As, Fe and Mn concentrations using rhizon samplers (product ID: 19.21.05, Rhizosphere Research Products, Wageningen, Netherlands). The rhizon sampler was lowered into the overlying water from the top of the core, essentially acting as a 5 cm long cylindrical filter (0.2 μm) and providing a more integrated sampling of the water column. Rhizons were triple rinsed with 10 ml of 2% nitric acid (HNO_3_) followed by a triple rinse of ultra pure water prior to use. Syringes used with the rhizons were pre-washed in a 2% HNO_3_ bath for a 48-hours, then triple rinsed with ultra pure water and air dried over night. Water samples obtained with the rhizons and syringes were placed in 15 mL trace metal vials, preserved with 2% HNO_3_, and stored in a refrigerator until analysis. Water samples of 10 mL were placed in 20 mL glass vials that were pre-washed with 2% HNO_3_ and combusted in a muffle oven at 425°C for 2 hours. The samples were stored with no preservatives in a refrigerator until analysis. For the SO_4_^2-^ water samples, 2 mL of overlying water was stored in 15 mL trace metal vials, preserved with 2% zinc acetate, and stored in a freezer until analysis.

### Porewater sampling

Porewater samples were collected for analysis of As, Mn, and Fe concentrations, and to assess changes in porewater concentrations with temperature treatments. Due to the fine particle sizes of Yellowknife Bay sediments, the syringes clogged, making porewater sampling impossible. Hence, porewater samples were only obtained from the two Lower Martin Lake sediment cores. To preserve the porewater samples as quickly as possible, 2% HNO_3_ was added directly to the syringe that was used to extract the porewater prior to sampling. A 0.45 μm disk filter was added onto the syringe to prevent sediments from entering the porewater sample. Each 3 mL sample of porewater was stored in a 15 mL trace metal vial in a refrigerator until analysis.

### Incubated sediment sampling

Once the experiment was completed, the sediments were collected from each core to analyze for bulk concentrations of metal(loid)s. The cores were sliced using a UWITEC sediment extruder (UWITEC GmbH, Mondsee, Austria). Because the high OM content of sediments and low mass of material available from Lower Martin Lake, the top 3 cm of each core was collected to obtain sufficient material for analysis. The two Yellowknife Bay cores were sliced into several intervals (the top 1 cm of sediment was collected as one sample, followed by 0.5 cm intervals of sediment slices to 5 cm depth).

### Chemical analysis

Water concentrations of As, Mn and Fe were measured by inductively coupled plasma mass spectrometry (ICP-MS) according to EPA method 200.8 at Taiga Laboratories (Yellowknife, Northwest Territories, Canada). Field blanks, travel blanks and field duplicates were collected for quality assurance and quality control (QA/QC) measures during the experiment. For the surface water sampling, a total of 21 QA/QC samples were taken: 8 field blanks, 6 travel blanks and 7 field duplicates. The average relative standard deviation (RSD) between duplicate samples for As, Mn and Fe were 1.5 ± 1%, 8 ± 17%, and 6 ± 9%. Of the 8 field blanks, each one was below the method detection limit (MDL) for As and Fe of 0.2 and 5.0 μg/L, respectively, but 6 of the 8 field blanks measured a Mn concentration around the MDL of 0.2 μg/L. Sample results for water Mn concentrations were not blank corrected because the average Mn concentration in the field blanks (0.2 ± 0.1 μg/L) was <10% of most water sample values.

Filtered water was analyzed for DOC and DN at RPC Laboratories (Fredericton, New Brunswick, Canada) according to APHA 5310 B, with a principal method of combustion and chemiluminescence by non-dispersive infrared (NDIR) detection. Filtered water was analyzed for SO_4_^2-^ by ion chromatography on a Dionex instrument at the Geochemistry Laboratory (Department of Earth and Environmental Sciences, University of Ottawa, Ottawa, ON, Canada). Sediments from the experimental cores were analyzed for acid-leachable element concentrations by ICP-MS following aqua-regia digestion at Bureau Veritas (Vancouver, British Columbia, Canada). Organic matter content was measured by loss on ignition (LOI) of sediment sub-samples in a muffle oven (550°C for 4 hours). Details on QA/QC for these chemical analyses are provided in the Supplementary Information (**S1 Text in S1 File**).

### Data analysis

All raw data for the experiment are provided in **S2 File.**

#### Measured flux

The fluxes of As, Fe, Mn from sediment to overlying under each temperature treatment were calculated for each core using the surface water masses of each element on the initial and final day of each temperature treatment (**Eq 1**). The mass of each metal(loid) was calculated using the measured concentration of each metal(loid) in the surface water samples multiplied by the volume of the water column measured in litres. The volume of overlying water was not replaced during the experiment, rather the total volume used to calculate the element mass took into account the small volume of water removed after each water sampling event. The total amount of water removed for water chemistry sampling during the experiment was <15% of the total volume. After flux calculation, regression analysis was used to examine the influence of temperature on metal(loid) flux.


$$\mathrm{Flux}\;(\mu g/\frac{m^{2}}{day})=[\frac{\left(\mathrm{Final}\;\mathrm{Mass}\;(\mu g)-\mathrm{Initial}\;\mathrm{Mass}(\mu g)\right)}{\left(\mathrm{Number}\;\mathrm{of}\;\mathrm{days}\;\mathrm{in}\;\mathrm{treatment}\right)}]\times {\left(\mathrm{Sediment}\;\mathrm{Surface}\;\mathrm{Area}\;m^{2}\right)}^{-1}$$ (1)

#### Theoretical flux using Fick’s first law

Theoretical estimates of temperature effects on As flux were calculated for Lower Martin Lake using porewater As concentrations and Fick’s first law of diffusion (**Eq** **2**). Fick’s first law relates element flux to the diffusion coefficient of the sediment, the formation resistivity factor, the porosity of the sediments and the measured concentration gradient [26].


$$Flux=-\left(\frac{D{}^{\circ}}{F}\times \varphi \times \frac{dc}{dz}\right)$$ (2)

The average porewater and surface water concentrations from measurements on the first and last days of the first treatment were used to calculate the As concentration gradients (dc/dz) for each Lower Martin Lake sediment core. Using Fick’s law, the theoretical estimates of temperature effects on sediment As flux were then calculated for each of the four temperature treatments. The theoretical As fluxes (reported as μg/m^2^/day) were plotted against observed flux results for each temperature treatment for comparison. Note this calculation did not account for temperature influences on biogeochemical reactions, as biological processes are not integrated into the flux calculations. More details on the Fick’s law calculations are provided in the Supplementary Information (**S2 Text in S1 File**).

## Results

### Sediment chemistry

**Fig 2:**
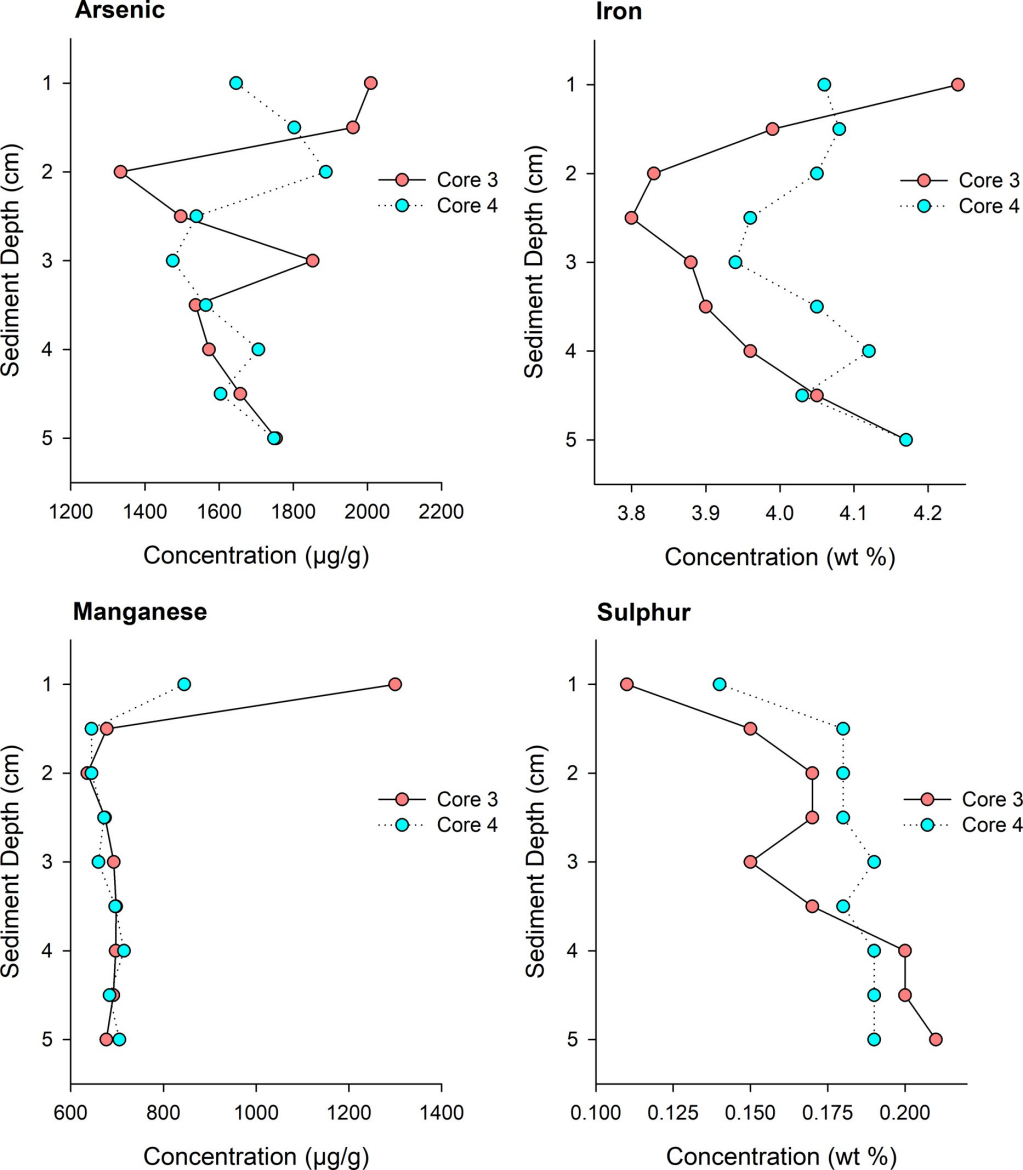
Sediment profiles of element concentrations. Profiles of As, Fe, Mn and S concentrations in the top 5 cm of sediment in the Yellowknife Bay experimental cores.

Sediment from each of the two study lakes differed in their chemical composition. Lower Martin Lake sediments had high OM content (70% LOI), while Yellowknife Bay sediments were OM-poor (10% LOI). Arsenic concentrations of the Lower Martin Lake sediment (average ± standard deviation = 822 μg/g ± 23 g/g, n = 2 slices) were roughly half of those of Yellowknife Bay sediment (1675 ±180 μg/g, n = 18 slices). Similarly, solid-phase concentrations of Mn and Fe were higher in Yellowknife Bay sediments compared with those of Lower Martin Lake (**S2 File**). In contrast, the sulphur content of Lower Martin Lake sediments was higher (1.4 ± 0.04%, n = 2) than in Yellowknife Bay sediments (0.2 ± 0.02%, n = 18), likely due to the contributions of organic sulphur in the OM-rich sediment. Concentrations of Mn were highest at 1 cm depth in the Yellowknife Bay sediment, while sulphur concentration was lowest at 1 cm and increased with depth (**Fig 2)**. The depth profiles of As and Fe were more variable in the Yellowknife Bay sediments with no clear patterns.

### Surface water chemistry

**Fig 3:**
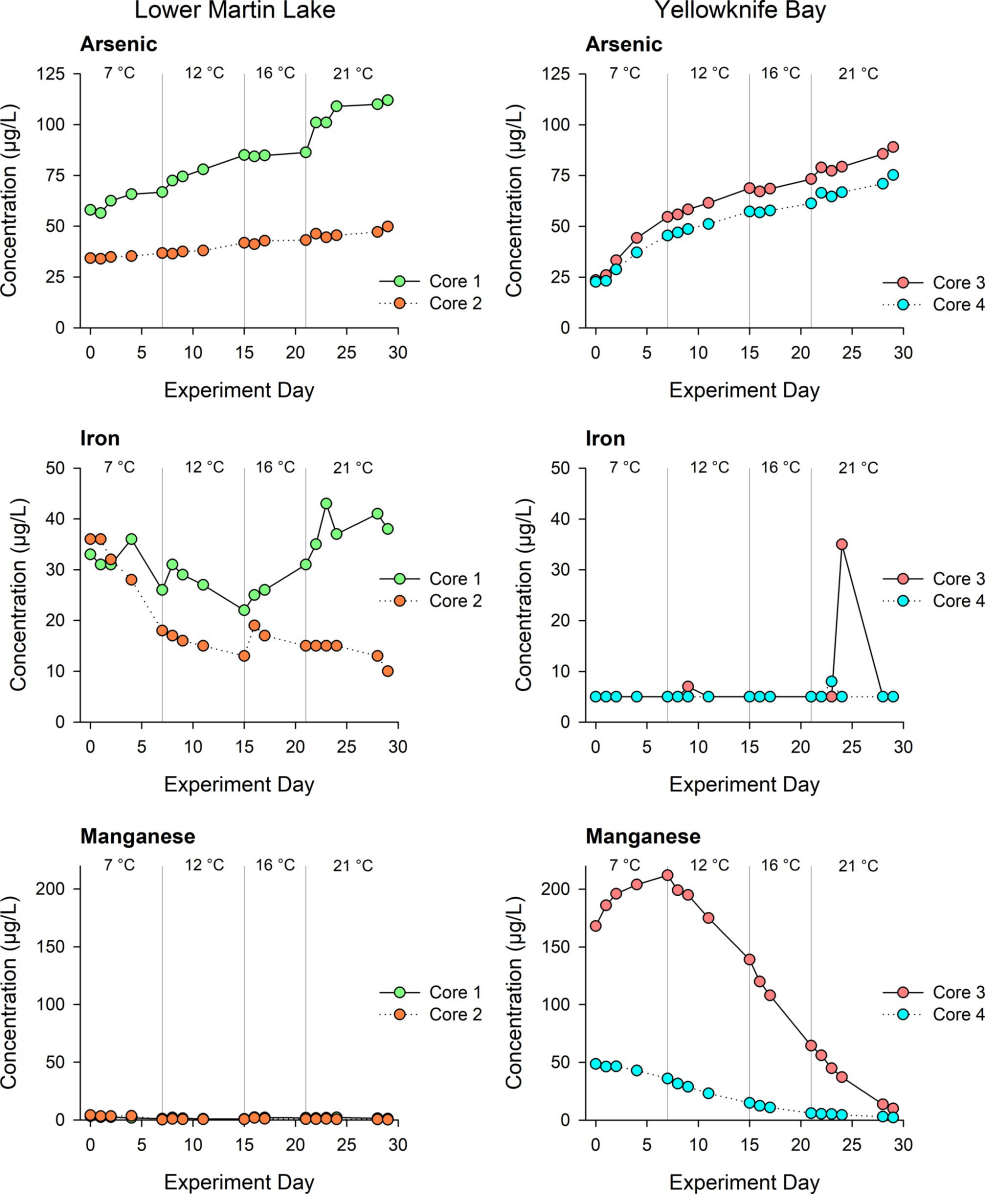
Time series of metal(loid) concentrations in overlying water. Metal(loid) concentrations of As, Fe, and Mn in overlying water in duplicate Lower Martin Lake and Yellowknife Bay cores throughout the 30-day experiment. Each vertical grey line represents the start date of a new temperature treatment. The precision of As, Fe, and Mn concentrations is estimated at < 9% based on the average RSD of seven duplicate measurements.

At the beginning of the experiment, dissolved As concentrations of the overlying water were 34 and 58 μg/L in the Lower Martin Lake cores and 23 and 24 μg/L in the Yellowknife Bay cores. The water As concentrations increased throughout the experiment in all sediment incubations, with approximately a two-fold increase in the Lower Martin Lake cores and a three-to-four-fold increase in the Yellowknife Bay cores (**Fig 3; S2 Table in S1 File**). One of the cores in Lower Martin Lake showed a greater increase in water As than the other, while both cores of Yellowknife Bay showed almost identical increasing trends. The water Fe concentrations remained relatively stable throughout the experiment in the Yellowknife Bay cores but fluctuated in the Lower Martin Lake cores. The water Mn concentrations of the Lower Martin Lake cores remained low and stable, whereas Yellowknife Bay cores showed an overall decrease, especially in core 3 which decreased from 212 to 10 μg/L throughout the experiment.

**Fig 4:**
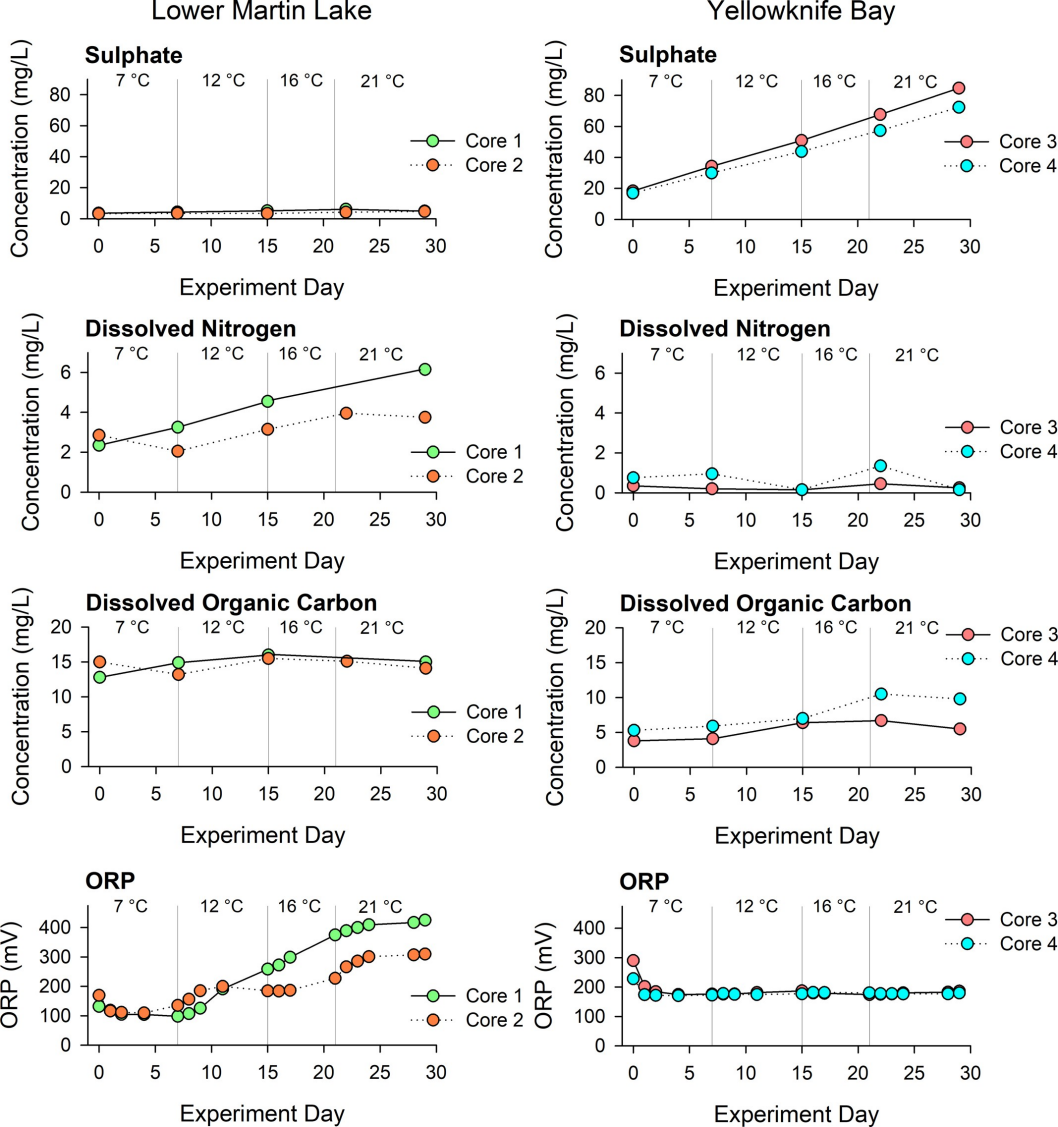
Time series of water quality measurements on overlying water. The concentrations of SO_4_^2-^, DN and DOC and ORP of overlying water in the duplicate Lower Martin Lake and Yellowknife Bay cores throughout the 30-day experiment. Each vertical grey line represents a new temperature treatment. The precision of SO_4_^2-^, DN, and DOC concentrations is estimated at < 10% based on RSD of three duplicates.

Water concentrations of SO_4_^2-^ in the Lower Martin Lake cores remained low and changed little throughout the experiment, while in Yellowknife Bay cores, SO_4_^2^- concentration increased linearly throughout the experiment from 17 to 85 mg/L (**Fig 4**). Dissolved nitrogen increased slightly in the Lower Martin Lake cores, whereas there was negligible change in the Yellowknife Bay cores. Dissolved organic carbon remained high and steady in the Lower Martin Lake cores (~15 mg/L) and slightly increased halfway through the experiment in one of the Yellowknife Bay cores. The ORP values were positive, indicated oxidizing conditions in the water column of all cores throughout the experiment. The ORP increased continuously in the Lower Martin Lake cores during the experiment (likely due to photosynthesis of algae at the sediment surface), while in the Yellowknife Bay cores, the ORP decreased slightly at the beginning though remained positive for the remainder of the experiment.

### Experimental sediment metal(loid) fluxes

**Fig 5:**
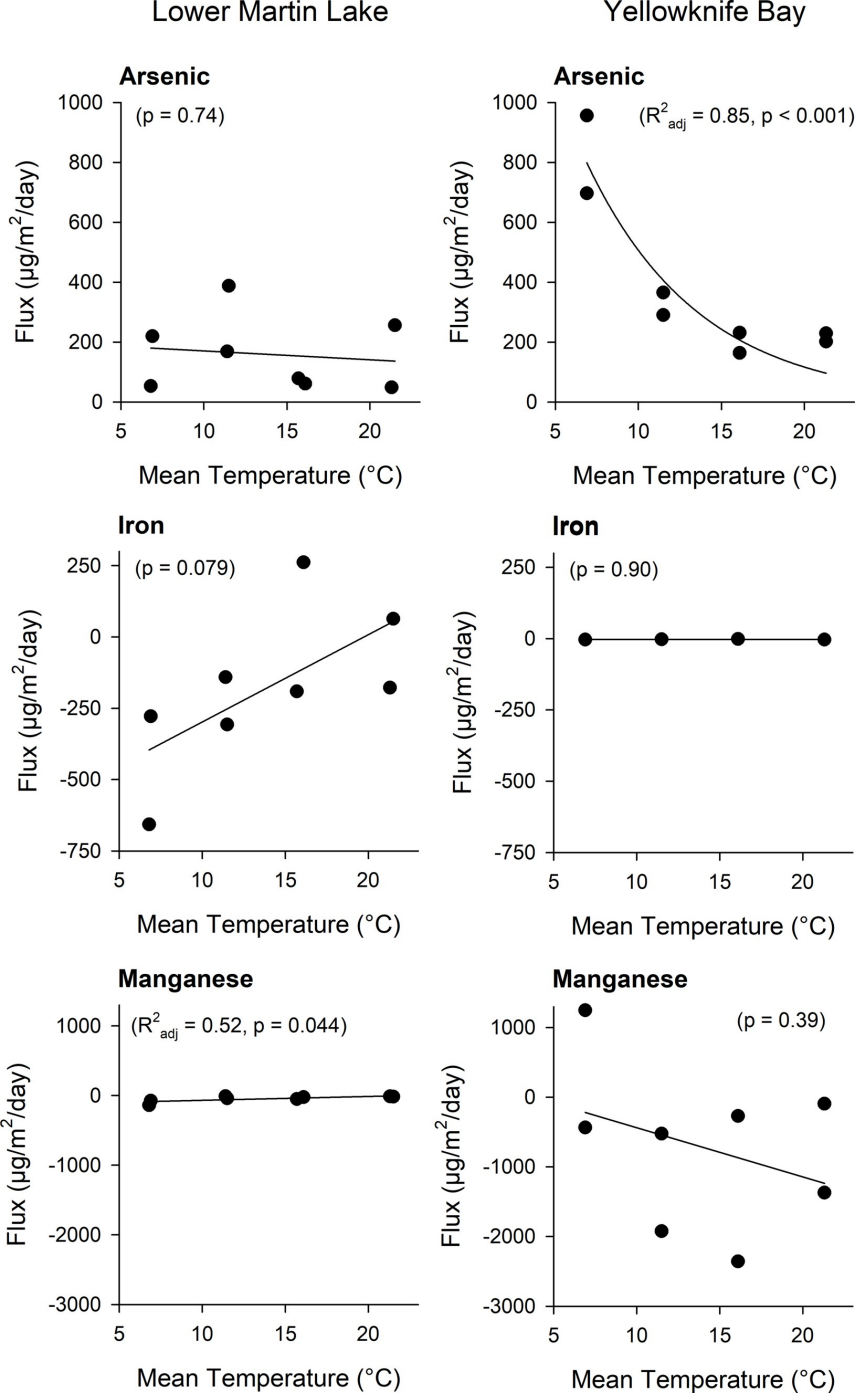
Experimental sediment metal(loid) fluxes in relation to temperature. Metal(loid) fluxes from sediment to overlying water of Lower Martin Lake and Yellowknife Bay cores incubated at different temperatures. There are two points for each temperature which are measured fluxes of duplicate cores for each temperature treatment. Coefficients of determination are provided for statistically significant regressions.

Duplicate measurements of metal(loid) fluxes from sediments of Lower Martin Lake and Yellowknife Bay showed little variation among temperature treatments over the course of the experiment (**Fig 5; S3 Table in S1 File**). The As fluxes to overlying water from organic-rich sediments of Lower Martin Lake did not change with temperature (p = 0.74), while the fluxes from organic-poor sediments of Yellowknife Bay were highest at the start of the experiment (with the coldest temperature treatment) then decreased to lower and constant fluxes for the warmer temperature treatments (R^2^_adj_ = 0.85, p < 0.001). Arsenic fluxes were, on average (± standard error), higher from Yellowknife Bay sediments (392 ± 100 μg/m^2^/day) than from sediments of Lower Martin Lake (159 ± 43 μg/m^2^/day). The fluxes of Fe from Lower Martin Lake sediment showed a marginal increase with temperature (p = 0.079) but on average remained negative, indicating settling out during the experiment. There was no Fe flux in the Yellowknife Bay cores. The Mn fluxes remained negative with increasing temperature in both the Lower Martin Lake and Yellowknife Bay cores.

### Theoretical estimates of temperature influence on sediment arsenic flux

For the Lower Martin Lake incubations, the average concentration (± standard deviation, n = 5) of porewater As at 1 cm below the sediment-water interface was 105 ± 14 μg/L in core 1 and 44 ± 10 μg/L in core 2. The average porewater Fe concentration during the experiment was 46 ± 25 μg/L in core 1 and 513 ± 245 μg/L in core 2. The average porewater concentration of Mn was 15 ± 19 μg/L in core 1 and 226 ± 81 μg/L in core 2. The large variation in porewater concentrations of As, Fe and Mn between cores indicated differences in concentrations gradients with overlying water and potentially different ORP conditions at 1 cm sediment depth.

Concentration gradients of As were observed between the porewater and overlying water of Lower Martin Lake sediment, although a larger gradient was observed in core 1 (-0.0521 μg/cm^4^) than in core 2 (-0.0027 μg/cm^4^). Porewater As concentrations at 1 cm depth remained relatively stable throughout the experiment in both core 1 (relative standard deviation = 13%) and core 2 (relative standard deviation = 22%). In addition, porewater As concentrations did not increase with warming during the experiment in either core (linear regressions, p > 0.05) (**S2 Fig in S1 File**).

**Fig 6:**
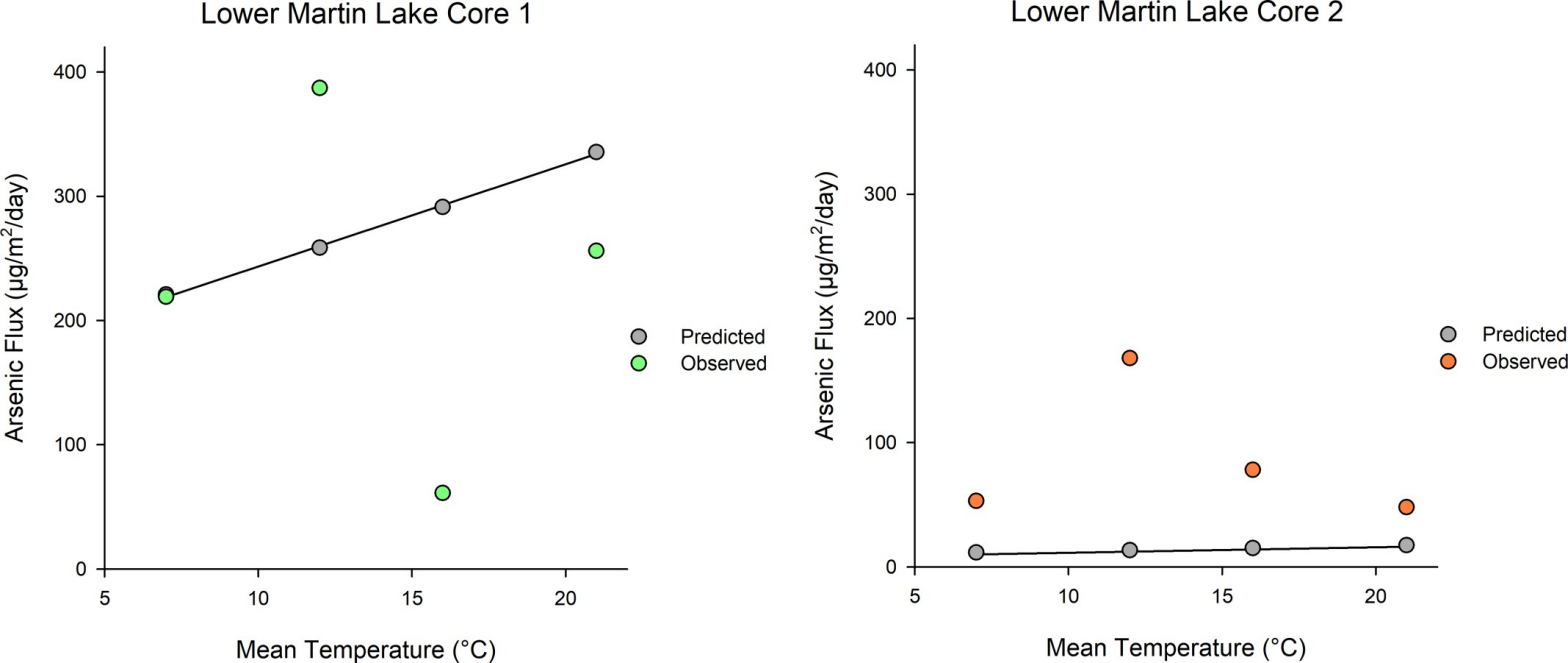
Predicted versus observed sediment arsenic fluxes in relation to temperature. Predicted As fluxes from Lower Martin Lake sediment (calculated using Fick’s law) for each temperature treatment in comparison to measured As fluxes in duplicate cores.

As shown in **Fig 6**, the theoretical As flux estimated by Fick’s Law predicted an increase by a factor of 1.6 between 7 and 21°C. The observed sediment As fluxes in the Lower Martin Lake cores were scattered near to the theoretical flux predictions. The average predicted flux (± standard deviation) across all temperature treatments was 277 ± 49 μg/m^2^/day for core 1 while the observed average flux was 231 ± 134 μg/m^2^/day. For core two, the average predicted flux was 14 ± 3 μg/m^2^/day while the observed average flux was 87 ± 56 μg/m^2^/day. Since the As concentration gradient between sediment porewater and overlying water in Core 2 was lower than in Core 1, the theoretical temperature effect was also lower.

## Discussion

### Arsenic flux from mining-contaminated lake sediment

Mining-contaminated sediment from Yellownife Bay and Lower Martin Lake showed that As remobilizes from sediment to overlying water, even when the water column is well-oxygenated. Experimental flux estimates from both Yellowknife Bay and Lower Martin Lake sediments (mean ± standard deviation = 392 ± 284 and 159 ± 122 μg/m^2^/day, respectively) were similar to field-based studies for those contaminated sites. Andrade et al. [15] estimated an As flux of 667–1033 μg/m^2^/day for north Yellowknife Bay sediments, while Van Den Berghe et al. [9] estimated an As flux of 168 μg/m^2^/day from Lower Martin Lake sediments. A lake in southern Canada with mining-contaminated sediments and well-oxygenated bottom waters also had comparable sediment As fluxes of 55–411 μg/m^2^/day [27]. In this study, Yellowknife Bay had twice the sediment As concentrations compared to Lower Martin Lake, which can explain the higher As fluxes in the Yellowknife Bay cores. Although the aerobic zone in near-surface sediment can function as a barrier to As diffusion across the sediment-water interface and near-surface Fe (oxy)hydroxides can absorb a considerable amount of As (3–4 wt %) [9,10], our experimental results are consistent with field-based observations of As diffusion from contaminated sediments of well-oxygenated lakes.

### Temperature influence on arsenic flux

Despite an increase in As concentration in the overlying water of all sediment cores throughout the 30-day experiment, there was no detected influence of warmer temperatures on the As flux for OM-rich and OM-poor sediment from two contaminated lakes. This finding was contrary to expectation given that sediment As flux can be enhanced under warmer temperatures. By controlling redox conditions of overlying water, through continuous aeration, and preventing temperature-driven microbial depletion of dissolved oxygen in the sediment surface layer at warmer temperatures, the experimental results indicate that temperature effects on As sediment flux, which have been reported in previous studies [16,20,28], may be due to indirect effects on microbial processing and alteration of redox gradients within near-surface sediments. The experimental results are consistent with *in situ* observations in Lower Martin Lake, where sediment As fluxes were highest at 4°C in winter when anoxia develops under ice, indicative of the over-riding effect of redox state [13,14].

Li et al. [20] conducted a batch experiment on mine-contaminated sediments collected from the Huangshui Creek in Hunan Province, China. In their experiment, sediment samples incubated under three temperature treatments of 8°C, 25°C and 37°C showed an increase in the release of As(III) from sediments at warmer temperatures, likely due to redox variations [20]. Multiple laboratory-based studies have shown that As mobility in saturated soils and sediments under reducing conditions is temperature dependent, as there was an increase in reduction of As(V) associated with an increase in temperature [17,29–31]. Although inorganic As speciation was not measured in this study, research conducted by Weber et al. [17] found, under anoxic conditions, temperature had an important influence on concentrations of dissolved Fe(II) and As(III). Similarly, Johnston et al. [28] incubated sediment from the Macleay River catchment (Australia) for 21 days at five temperature treatments at 8°C, 14°C, 20°C, 26°C and 32°C. Their study showed that temperature had a negligible impact on As mobility under oxic conditions, but that temperature had a indirect influence on reducing conditions through enhanced microbial respiration rates [28].

Typically, as temperature increases so does microbial respiration, resulting in a thinning of the sediment oxic layer at the surface. The subsequent upward migration of the Fe(III) redoxcline leads to the reductive dissolution of Fe (oxy)hydroxides and a release of bound As [16,32]. In this experiment, we infer that the thickness of the oxic boundary was maintained through continuous aeration of the overlying water, which limited the dissolution of Fe and Mn (oxy)hydroxides. Throughout the experiment, there was little variation in Fe and Mn concentrations in overlying waters and sometimes a negative flux indicating settling out with increasing temperature. Other indicators that an oxic boundary was maintained at the sediment surface through the experiment were low temporal variation in porewater As concentration (at 1 cm depth in the Lower Martin Lake cores), and lower % sulphur in the top 1 cm of Yellowknife Bay sediment (likely due to oxidation of sulphides; see additional explanation below).

### Theoretical estimates of sediment as diffusion

Theoretical As fluxes (which were calculated using Fick’s law and porewater to surface water concentration gradients for the sediment cores from Lower Martin Lake) predicted relatively small temperature effects. Flux rates were predicted to increase by a factor of 1.6 between 7 and 21°C, leading to an estimated maximum increase of 221 to 335 μg/m^2^/day in core 1 and 11 to 17 μg/m^2^/day in core 2. This increase is relatively small compared with differences between cores and lake sediment types in the experiment as well as relative to maximum As fluxes of 1,302 to 10,411 μg/m^2^/day from sediments reported in the literature [15,16,27]. Note Fick’s law assumes steady-state conditions (i.e. only represents diffusion along a concentration gradient), therefore any chemical processes such as changes in redox state at the sediment boundary or altered adsorption on Fe (oxy)hydroxides are not accounted for in the calculation. Fick’s law calculations and measurements of change in As concentration of overlying water provided two different ways to estimate temperature influences on As flux in this study. Overall, both methods indicated similar estimates of As flux, which support the validity of the experimental results.

### Experimental design

Potential biases from the experimental design were considered in the interpretation of findings. The short duration for each temperature treatment (i.e. 7 days) may not have provided sufficient time for temperature effects on microbial activity and subsequent effects on processes that could enhance As desorption. Nevertheless, no enhancement of As flux was observed when also considering the entire 30 day duration of the experiment (i.e., between the coldest and warmest treatments at the start and end of the experiment). Our results contrast with other experimental incubation studies that observed temperature effects on sediment As flux over a similar time interval of less than 30 days [16,20]. Further, the short-term warming tested in the experiment is consistent with typical water temperature regimes in the subarctic lakes. Unpublished high frequency measurements of water temperature in both Yellowknife Bay and Lower Martin Lake showed large oscillations during the open water season, with multiple periods of warming often only lasting for 1–2 weeks before cooling (**S3 and S4 Figs in S1 File**). Thus for these systems, an experimental design involving long incubations at a single, stable temperature would not reflect actual temperature regimes of the contaminated sediments. The subarctic lakes in this study are different ecosystems compared with those investigated in other published research on temperature interactions with As at lower latitudes [16,17,28].

Another consideration was the use of mixed sediment for the Yellowknife Bay cores, which would have altered the layering and mineral composition relative to natural conditions. In particular, sediment mixing brought As-sulphide minerals from deeper layers to the surface, where oxidation would have occurred with potential release of As to overlying water. The increase in water SO_4_^2-^ of Yellowknife Bay cores during the experiment was likely due to the oxidation of sulphides at the sediment surface, which did not appear to have equilibrated by the end of the experiment. This process may not typically occur under natural conditions without sediment disturbance. While the contribution of As-sulphide mineral oxidation to As flux could not be determined, recent measurements of As mineralogy in sediment of Yellowknife Bay indicated that realgar (As_4_S_4_) is generally present in low amounts, with low contributions to total solid-phase As concentrations [24]. Microbe-mediated dissolution of realgar may also be temperature dependent [33]. The similarity of our experimental As flux estimates with those of intact undisturbed cores [15] suggest that As-sulphide oxidation did not have an important influence on the Yellowknife Bay sediment flux results.

### Implications of climate change for arsenic contamination in the study area

The western Canadian Arctic has undergone the greatest warming among regions in Canada during the 20^th^ century and continued warming is expected with climate change [34]. For Yellowknife, the mean annual temperature has increased by ~3°C between 1956 and 2016 [35]. Our experimental results suggest that warmer lake water and sediment temperatures will not directly affect sediment As fluxes in well-oxygenated lakes, at least on short time scales. However, altered environmental conditions from climate warming may have an indirect effect on As mobility in mining contaminated lakes of the Yellowknife area. Predictive models have suggested that greater water column stratification and a decrease in mixing will lead to oxygen depletion and the development of anoxic areas near the sediment-water boundary [36,37]. Increased periods of water column anoxia near the sediment-water boundary has been shown to increase As fluxes from contaminated sediments [32]. Increased primary production in subarctic lakes experiencing climate warming could also alter dissolved oxygen concentrations by leading to greater OM accumulation and stimulation of microbial metabolism within lake sediments. Climate change is altering the amount of precipitation within the Arctic, though less so in the study area [34]. Changes in hydrology, seasonal temperatures, and carbon flux within lakes all have an impact on biogeochemical cycles within aquatic ecosystems, including for As [14,38]. As warming continues, long-term monitoring of contaminated lakes in the Yellowknife region should track ecosystem change to evaluate potential implications of temperature-mediated effects on sediment redox conditions, OM cycling and water dissolved oxygen concentrations for As remobilization from sediments.

## Conclusions

This detailed experiment for two subarctic lakes showed temperature had a negligible direct impact on the flux of As from organic-rich and organic-poor sediments contaminated with As, under oxidizing conditions in the water column. This finding of no temperature effect is an important contrast to other research because it demonstrates temperature effects may be system-specific. Previously reported effects of temperature on sediment As fluxes in other studies may be due to indirect effects of temperature on biological oxygen demand and dissolved oxygen/redox conditions in the sediment surface layer, which impact desorption and dissolution of As minerals in near-surface sediments. Future research should investigate how climate-mediated changes to sediment biological oxygen demand and enhanced carbon cycling may impact As mobility in contaminated subarctic lakes.

## Supporting information

S1 File Appendix. This file contains supplementary figures, tables and equations on experimental methods or results, as well as *in situ* temperature profiles of the study lakes.
(DOCX)

S2 File Raw data. This file contains element concentrations of overlying water, sediment and porewater generated during the experimental incubation of contaminated lake sediments.
(XLSX)
