# Supplementary material for: Experimental investigation of short-term warming on arsenic flux from contaminated sediments of two well-oxygenated subarctic lakes
Source: PLoS One. 2022 Dec 21;17(12):e0279412. doi: 10.1371/journal.pone.0279412 (PMC9770359; doi:10.1371/journal.pone.0279412)
Supplement: S1 File — This file contains supplementary figures, tables and equations on experimental methods or results, as well as in situ temperature profiles of the study lakes. (DOCX) [file pone.0279412.s001.docx]

**S1_File. Appendix**

**Experimental investigation of short-term warming on arsenic flux from contaminated sediments of two well-oxygenated subarctic lakes**

Brittany C. Astles^1^, John Chételat^2*^, Michael J. Palmer^3^, Jesse C. Vermaire^1^

^1^ Geography and Environmental Studies, Carleton University, Ottawa, Canada

^2^ National Wildlife Research Centre, Environment and Climate Change Canada, Ottawa, Canada

^3^ North Slave Research Centre, Aurora Research Institute, Yellowknife, Canada

* Corresponding Author:

Email: [john.chetelat@ec.gc.ca](mailto:john.chetelat@ec.gc.ca)

Contents

[**Figure S1:** Experimental set-up for incubated sediment cores from Yellowknife Bay of Great Slave Lake and Lower Martin Lake. 3](#_Toc114641466)

[**Table S1:** Experimental sampling schedule for surface water and porewater collection. 4](#_Toc114641467)

[**Table S2:** Chemical characteristics of overlying water during the sediment incubation experiment. 5](#_Toc114641468)

[**Text S1:** QA/QC details for chemical analysis of dissolved organic carbon, dissolved nitrogen and sulphate in water, and metal(loid)s in sediment 6](#_Toc114641469)

[**Text S2.** Calculation of sediment arsenic fluxes in Lower Martin Lake cores using Fick’s law of diffusion 7](#_Toc114641470)

[**Table S3:** Metal(loid) flux estimates (μg/m^2^/day) from Yellowknife Bay and Lower Martin Lake sediments, averaged across temperature treatments. 9](#_Toc114641471)

[**Figure S2:** Porewater arsenic concentrations in the Lower Martin Lake cores throughout the experiment 10](#_Toc114641472)

[**Figure S3.** Water temperature of Lower Martin Lake during the open water season of 2018. 11](#_Toc114641473)

[**Figure S4.** Heat maps of water column temperatures in Yellowknife Bay during the open water season in 2014 and 2015. 12](#_Toc114641474)


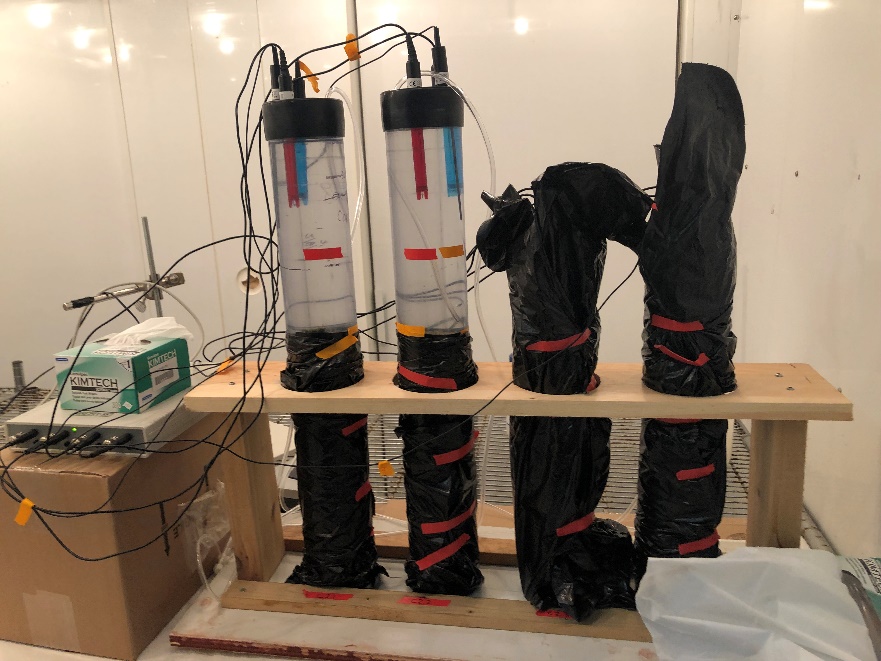


**Figure S1:** Experimental set-up for incubated sediment cores from Yellowknife Bay of Great Slave Lake and Lower Martin Lake. Note the opaque plastic bags covering the sediments within each core to prevent algal growth except at the surface of the Lower Martin Lake sediment. Also, note the cap of each core with the wiring and drilled ports for the sensors for continuous measurement of temperature, pH and redox potential, and piping for an oxygen bubbler.

## **Table S1:** Experimental sampling schedule for surface water and porewater collection.

| Temperature Treatment | Average Realized Temperature | Sampling Day | | | |
| --- | --- | --- | --- | --- | --- |
|  |  | Surface Water As, Mn and Fe | Surface Water DN and DOC | Surface Water SO_4_^2-^ | Porewater  As, Mn and Fe |
| Treatment 1 at $5 ℃$ | 7 $℃$ | 0, 1, 2, 4, 7 | 0, 7 | 0, 7 | 0, 7 |
| Treatment 2 at 10$℃$ | 12$℃$ | 8, 9, 11, 15 | 15 | 15 | 15 |
| Treatment 3 at $15 ℃$ | 16$℃$ | 16, 17, 21 | 22 | 22 | 22 |
| Treatment 4 at 20 $℃$ | 21$℃$ | 22, 23, 24, 28, 29 | 29 | 29 | 29 |

## **Table S2:** Chemical characteristics of overlying water during the sediment incubation experiment.

|  | Metal(loid) | Mean | Standard Deviation ($\pm$) | Range |
| --- | --- | --- | --- | --- |
| Lower Martin Lake cores | As ($\mu g/L)$ | 61.6 | 25.3 | 33.9-112.0 |
|  | Fe ($\mu g/L)$ | 25.6 | 9.4 | 10.0-43.0 |
|  | Mn ($\mu g/L)$ | 1.5 | 1.0 | 0.2-4.1 |
|  | SO_4_^2-^ ($m$g/$L$) | 4.2 | 0.8 | 3.2-6.0 |
|  | DN ($mg/L)$ | 3.5 | 1.2 | 2.1-6.2 |
|  | DOC ($mg/L)$ | 14.5 | 1.1 | 12.8-16.0 |
|  | ORP ($mV$) | 225 | 107 | 98-426 |
| Yellowknife Bay cores | As ($\mu g/L)$ | 56.6 | 18.6 | 22.6-89 .0 |
|  | Fe ($\mu g/L)$ | 6.0 | 5.2 | 5.0-35.0 |
|  | Mn ($\mu g/L)$ | 73.4 | 74.3 | 2.2-212.0 |
|  | SO_4_^2-^ ($m$g/$L$) | 47.6 | 23.1 | 17.0-84.7 |
|  | DN ($mg/L)$ | 0.6 | 0.4 | 0.2-1.4 |
|  | DOC ($mg/L)$ | 6.5 | 2.2 | 3.8-10.5 |
|  | ORP ($mV$) | 183 | 21 | 171-290 |

## **Text S1:** QA/QC details for chemical analysis of dissolved organic carbon, dissolved nitrogen and sulphate in water, and metal(loid)s in sediment

*Dissolved nitrogen and dissolved organic carbon:* A total of 2 field blanks, 2 travel blanks and 3 duplicates were analyzed for both dissolved nitrogen (DN) and dissolved organic carbon (DOC). There was contamination found in the field blanks for both DN (mean = 0.45 mg/L, range = 0.3-0.65 mg/L) and DOC (mean = 1.9 mg/L, range = 1.5-2.3 mg). Sample results for DN and DOC were therefore corrected by subtracting the average concentrations in the field blanks. The average RSD of the duplicate samples was 9 ± 9 % for DN and 7 ± 3 % for DOC.

*Sulphate:* The average RSD for three SO_4_^2-^ duplicate measurements was 0.5 ± 0.6 %. Two field blanks were below or at the MDL of 0.07 mg/L.

*Metal(loid)s in sediment:* The QA/QC included 3 blanks, a duplicate of one of the sediment samples, and analysis of certified reference materials (CRMs) to estimate recovery of elements. All 3 blanks were below the MDLs for Mn, Fe and S, while one of the blanks had an As concentration just above the 0.1 μg/g MDL at 0.2 μg/g. The duplicate RSD showed high precision for Mn, Fe, As, and S of 1.7 %, 0.9 %, 1.0 % and 0 %. The percent recovery of Mn, Fe, As, and S ranged from 92 - 100 % for the OREAS 262 CRM (Ore Research & Exploration Pty Ltd, Australia). Two PACS-2 Marine Sediment CRMs (National Research Council of Canada, Canada) with recoveries of As, S, Mn, and Fe at 90 %, 95 %, 55 %, and 74 %, respectively. Low recoveries of Mn and Fe were likely due to partial digestion with aqua-regia.

## **Text S2.** Calculation of sediment arsenic fluxes in Lower Martin Lake cores using Fick’s law of diffusion

Theoretical estimates of temperature effects on As flux were calculated for Lower Martin Lake using porewater As concentrations and Fick’s first law of diffusion (Equation S1). Fick’s first law relates element flux to the diffusion coefficient of the sediment, the formation resistivity factor, the porosity of the sediments and the measured concentration gradient (Lavery et al. 2001).

**Equation S1:** Fick's first law

$$Flux=-\left( \frac{D^{\circ}}{F}\times\varphi\times\frac{dc}{dz} \right)$$

The diffusion coefficient ($D^{^{\circ}}$) is dependent on the viscosity of the water at the ambient temperature (**Equation S2**). The ambient temperatures were set to mean temperature, in degrees Celsius, for each temperature treatment, represented by the coefficient T$^{\circ}$. At 25 °C, the diffusion coefficient of arsenate is 8.75$\times{10}^{-6}$cm^2^/s with a viscosity of 1.42 mPa/s (Tanaka et al. 2013). The standard viscosity coefficient ($V^{\circ}, mPa/s$) of water at 25 °C was used to interpolate the viscosity coefficient for each ambient temperature (**Equation S3**). These viscosity coefficients are then used to calculate the diffusion coefficient for each ambient temperature.

**Equation S2:** Diffusion coefficient equation

$$Diffusion \left( D^{\circ} \right)=\frac{\left[ \frac{D^{\circ} at 25 ℃\times Viscosity at 25 ℃}{25 ℃+273.15 ^{\circ}K}\times\left( T^{\circ}+273.15 ^{\circ}K \right) \right]}{Viscosity at T^{\circ}}$$

**Equation S3:** Water viscosity coefficient at given temperature

$$V^{\circ} =1000\times\left( \frac{2.414}{{10}^{5}} \right)\times{10}^{\left( \left( \frac{247.8}{T^{\circ} +273.15 ^{\circ}K} \right)-140 \right)}$$

The porosity coefficient ($\varphi$) is unitless and required the sediment bulk density and particle density of the sediment (**Equation S4**) from Lower Martin Lake. The measured average bulk density for Lower Martin Lake was calculated to be 0.01 g/cm^3^ based on the two 0-3 cm surface samples. The bulk density is low because sediment of Lower Martin Lake is rich in OM and benthic algae. The particle density of OM (0.8 g/cm^3^) was assumed for the Lower Martin Lake sediment. The sediment porosity ($\varphi$) was calculated to be 0.98. The Formation coefficient ($F$) is unitless and was calculated for organic rich sediments with high porosity, $\varphi$> 0.7, to be $F$= 1.04.

**Equation S4:** Porosity equation

$$\varphi=1-\left( \frac{Sediment Bulk Density}{Average Particle Density} \right)$$

The average porewater and surface water concentrations from measurements on the first and last days of the first treatment were used to calculate the concentration gradients (dc/dz) for each Lower Martin Lake sediment core and to calculate the theoretical effects of the temperature treatment on As flux (**Equation S5**). Using Fick’s law, the theoretical estimates of temperature effects on the As flux of Lower Martin Lake were calculated for each of the four temperature treatments. The distance of the gradient was 1 cm.

**Equation S5:** Concentration gradient

$$\frac{dc}{dz} (\mu g/{cm}^{4})=\frac{\frac{Surface Water \left[ As \right]\left( \frac{\mu g}{L} \right)-Pore water \left[ As \right](\mu g/L)}{distance of gradient (cm)}}{1000 {cm}^{3}/L}$$

The theoretical As flux was calculated for each core (reported as g/m^2^/day) and plotted against observed results for each temperature treatment for comparison. Note this calculation did not account for temperature influences on biogeochemical reactions, as biological processes are not integrated into the flux calculations.

References:

Lavery PS, Oldham CE, Ghisalberti M. The use of Fick's First Law for predicting porewater nutrient fluxes under diffusive conditions. Hydrological Processes. 2001;15(13):2435-51. doi: <https://doi.org/10.1002/hyp.297>.

Tanaka M, Takahashi Y, Yamaguchi N, Kim K-W, Zheng G, Sakamitsu M. The difference of diffusion coefficients in water for arsenic compounds at various pH and its dominant factors implied by molecular simulations. Geochimica et Cosmochimica Acta. 2013;105:360-71. doi: <https://doi.org/10.1016/j.gca.2012.12.004>.

## **Table S3:** Metal(loid) flux estimates (μg/m^2^/day) from Yellowknife Bay and Lower Martin Lake sediments, averaged across temperature treatments.

|  | **Lower Martin Lake** | | **Yellowknife Bay** | |
| --- | --- | --- | --- | --- |
| **Metal(loid)** | **Mean (**$\boldsymbol{\pm Standard}$  $\boldsymbol{Deviation)}$ | **Range** | **Mean (**$\boldsymbol{\pm Standard}$  $\boldsymbol{Deviation)}$ | **Range** |
| As | 159 $\pm$ 122 | 48 to 387 | 392 $\pm$ 284 | 164 to 956 |
| Fe | -178 $\pm$ 270 | -657 to 261 | -3 $\pm$ 1 | -4 to -2 |
| Mn | -46 $\pm$ 44 | -139 to -10 | -717 $\pm$ 1143 | -2359 to 1247 |


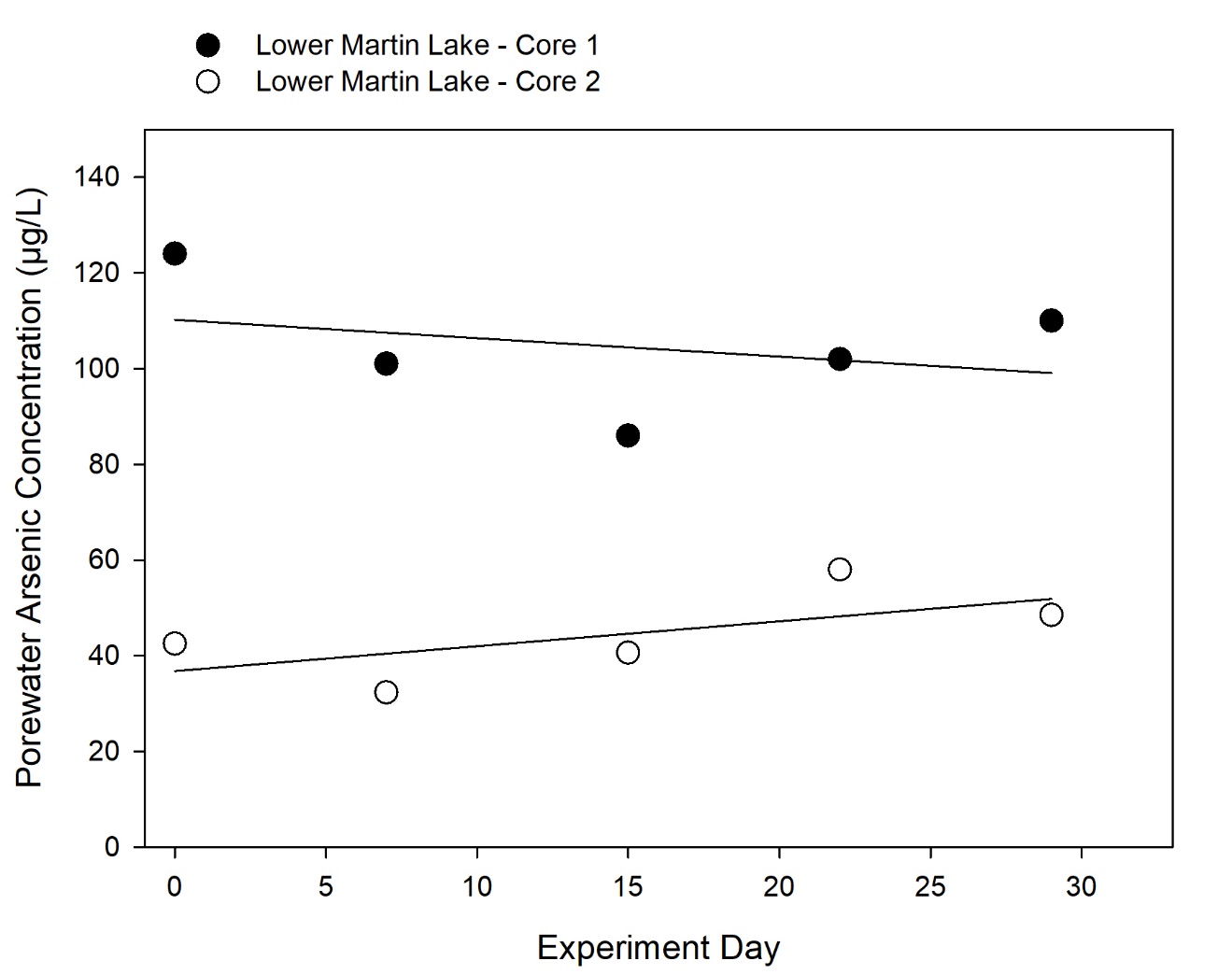


**Figure S2:** Porewater arsenic concentrations in the Lower Martin Lake cores throughout the experiment. Note there was no linear increase in porewater arsenic concentration at 1 cm depth for either core (p > 0.05).


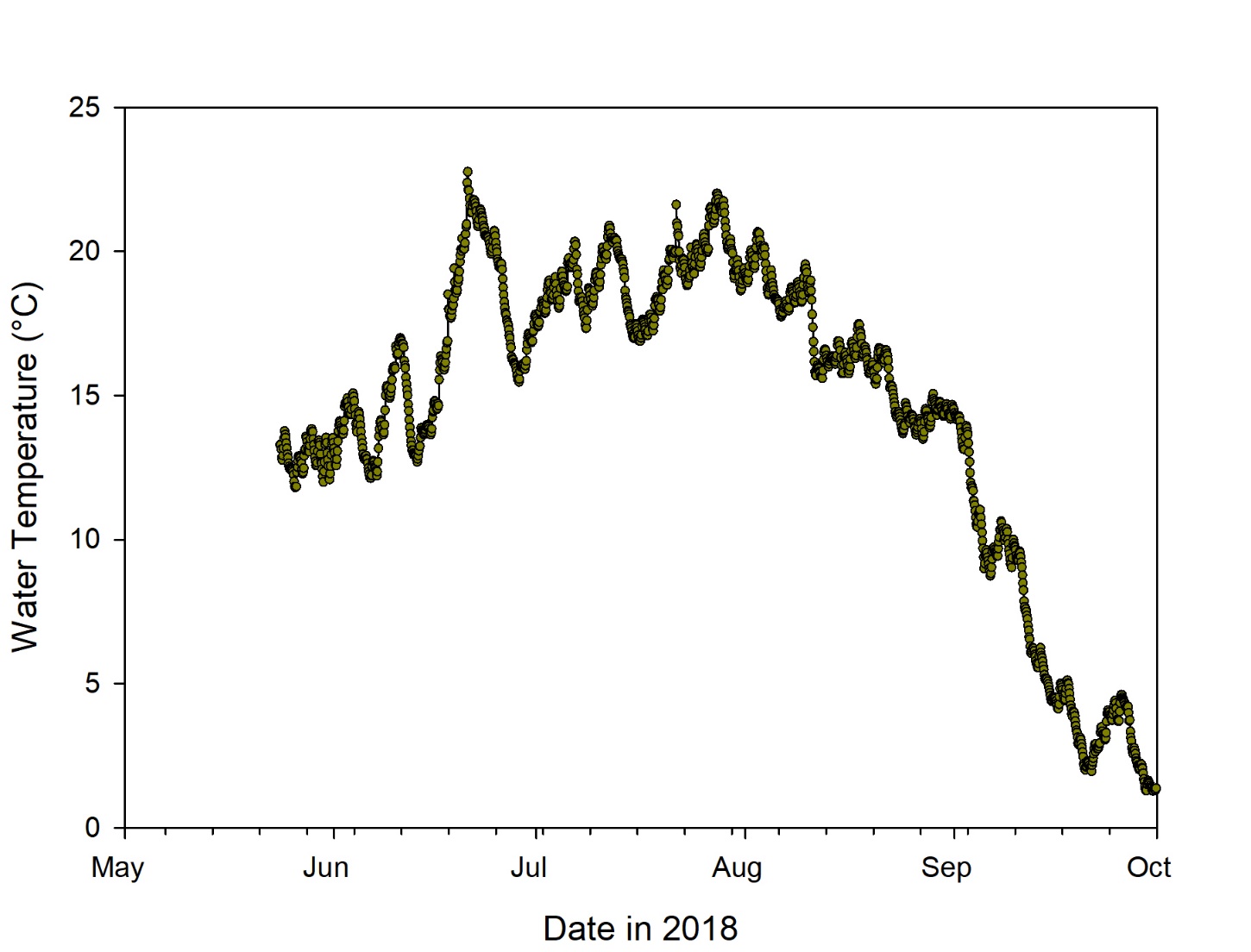


**Figure S3.** Water temperature of Lower Martin Lake during the open water season of 2018. High frequency measurements were taken every 2 hours using an ibutton temperature logger suspended in the water column at 1.75 m depth (maximum depth = 2.9 m).

**Figure S4.** Heat maps of water column temperatures in Yellowknife Bay during the open water season in 2014 and 2015. High frequency measurements were taken every 50 minutes using ibutton temperature loggers suspended at 1 m intervals in the water column starting at 2 m depth in 2014 (upper panel) and starting at the water surface (lower panel) in 2015. The maximum depth at the site in north Yellowknife Bay was 11 m.
